# Addition of 25-hydroxyvitamin D levels to the Deyo-Charlson Comorbidity Index improves 90-day mortality prediction in critically ill patients

**DOI:** 10.1186/s40560-016-0165-0

**Published:** 2016-06-17

**Authors:** Bisundev Mahato, Tiffany M. N. Otero, Carrie A. Holland, Patrick T. Giguere, Ednan K. Bajwa, Carlos A. Camargo, Sadeq A. Quraishi

**Affiliations:** Department of Anesthesia, Critical Care and Pain Medicine, Massachusetts General Hospital, 55 Fruit Street, GRJ 402, Boston, MA USA; Harvard Medical School, Boston, MA USA; Department of Anesthesiology & Perioperative Care, University of California – Irvine, Orange, CA USA; Tufts University School of Medicine, Boston, MA USA; Department of Medicine, Massachusetts General Hospital, Boston, MA USA; Department of Emergency Medicine, Massachusetts General Hospital, Boston, MA USA; Department of Medicine, Harvard Medical School, Boston, MA USA; Department of Epidemiology, Harvard School of Public Health, Boston, MA USA; Department of Anaesthesia, Harvard Medical School, Boston, MA USA

**Keywords:** Vitamin D, 25-hydroxyvitamin D, Mortality, ICU

## Abstract

**Background:**

The Deyo-Charlson Comorbidity Index (DCCI) has low predictive value in the intensive care unit (ICU). Our goal was to determine whether addition of 25-hydroxyvitamin D (25OHD) levels to the DCCI improved 90-day mortality prediction in critically ill patients.

**Methods:**

Plasma 25OHD levels, DCCI, and Acute Physiology and Chronic Health Evaluation II (APACHE II) scores were assessed within 24 h of admission in 310 ICU patients. Receiver operating characteristic curves of the prediction scores, without and with the addition of 25OHD levels, for 90-day mortality were constructed and the areas under the curve (AUC) were compared for equality.

**Results:**

Mean (standard deviation) plasma 25OHD levels, DCCI, and APACHE II score were 19 (SD 8) ng/mL, 4 (SD 3), and 17 (SD 9), respectively. Overall 90-day mortality was 19 %. AUC for DCCI vs. DCCI + 25OHD was 0.68 (95 % CI 0.58–0.77) vs. 0.75 (95 % CI 0.67–0.83); *p* < 0.001. AUC for APACHE II vs. APACHE II + 25OHD was 0.81 (95 % CI 0.73–0.88) vs. 0.82 (95 % CI 0.75–0.89); *p* < 0.001. There was a significant difference between the AUC for DCCI + 25OHD and APACHE II + 25OHD (*p* = 0.04) but not between the AUC for DCCI + 25OHD and APACHE II (*p* = 0.12).

**Conclusions:**

In our cohort of ICU patients, the addition of 25OHD levels to the DCCI improved 90-day mortality prediction compared to the DCCI alone. Moreover, the predictive capability of DCCI + 25OHD was comparable to that of APACHE II. Future prospective studies are needed to validate our findings and to determine whether the use of DCCI + 25OHD can influence clinical decision-making.

## Background

Mortality prediction scores are widely used in the intensive care unit (ICU) to predict the likelihood of survival from critical illness. These scores often assist healthcare providers in their discussions with patients and their family members about realistic expectations regarding ICU care and may be helpful in setting more patient-centered goals, assessing resource utilization, and providing higher quality of care [1]. Mortality prediction scores are also widely used by researchers to risk adjust for severity of illness, especially in multivariable regression models [2, 3].

While several mortality prediction scores for critical illness have been developed and are in current use, the Acute Physiology and Chronic Health Evaluation II (APACHE II), first published in 1985 [4], is the most commonly used worldwide [5]. However, APACHE II score calculations require measurement of specific physiological parameters within 24 h of ICU admission—this can be challenging, especially if a robust electronic medical record system is not available. Moreover, given that the score is heavily based on post-admission physiological assessments, it is not readily available at the time of admission, and it is of limited value for decision-making at the outset of critical illness.

Given the challenges of the APACHE II calculation (and for other similar mortality prediction models heavily influenced by physiologic assessments), the use of scoring systems based largely on medical history (i.e., comorbidities), despite being less accurate, are growing in popularity in the ICU literature [6, 7]. Among these, the Deyo-Charlson Comorbidity Index (DCCI) has been reported most frequently in studies of critical illness [8]. The DCCI [9], although modified from the original Charlson Index [6], continues to have a low predictive value for mortality in ICU patient cohorts [10–14]. Various modifications of either the original Charlson Index or DCCI have been made to improve their predictive values. These modifications have typically involved the use of administrative data [15–18], and they are not specific to ICU patients. And although the inclusion of several laboratory test results in the APACHE II scoring method is integral to its superior performance over the DCCI for mortality prediction in ICU patients [19], the impact of adding easily measurable or readily available biomarkers to improve the predictive capabilities of the DCCI in ICU patients has been largely underexplored [20].

Mounting evidence suggests that vitamin D status is associated with mortality in critically ill patients [21–27]. Indeed, assessment of vitamin D status in ICU patients is becoming increasingly common in the USA and worldwide [21, 28, 29]. In general, serum 25-hydroxyvitamin D (25OHD) levels are considered the best marker of total body vitamin D status [30]. Therefore, our goal was to determine whether the addition of 25OHD levels, measured at the outset of critical illness, to the DCCI improves mortality prediction in ICU patients.

## Methods

We performed a retrospective analysis of the data from an ongoing prospective cohort study designed to assess vitamin D status in critically ill patients. A subset of these patients was previously described in studies that investigated the association of vitamin D status with duration of mechanical ventilation and 90-day mortality in ICU patients [25, 31]. For the present study, subjects were recruited from three, 18-bed ICUs (1 surgical, 1 medical, and 1 mixed surgical/medical) at the Massachusetts General Hospital (MGH), in Boston, MA. The ICUs received admissions from all surgical and medical services except for Cardiac Surgery and Cardiology, respectively. All subjects were enrolled between 06/01/2012 and 05/30/2015. MGH is a 1052-bed, teaching hospital and a level-one trauma center, which serves a diverse population in and around Eastern Massachusetts. The Partners Human Research Committee (Institutional Review Board) approved the study protocol.

### Inclusion and exclusion criteria

All adult males and females, ≥18 years of age, and who were expected to require at least 48 h of critical care (as determined by the treating ICU team) were deemed eligible to participate. Informed consent was obtained either directly from subjects or appropriate healthcare surrogates. Subjects were only included in the study if blood samples to assess vitamin D status could be obtained within 24 h of admission to the ICU. Exclusion criteria included a known history of anemia at the time of ICU admission (defined as hematocrit <25 %), pregnancy or immediate post-partum status, and history of vitamin D supplementation ≥4000 IU/day. To minimize confounding from either partially treated, new-onset illness, or chronic illnesses, subjects were also excluded if they were transferred from another ICU or had been in an ICU within 1 year of the most current admission. Patients expected to transition to “comfort only measures” were also excluded.

### Blood sample processing and biomarker assays

Following informed consent, fresh blood was acquired from an indwelling arterial or central venous catheter and was collected directly into an EDTA-containing tube (lavender top). The sample was immediately stored on ice and then centrifuged within 30 min to separate out plasma. All samples were centrifuged at 2300 rpm for 15 min at a temperature of 4 °C. The separated plasma was immediately transferred to polypropylene tubes and stored at −80 °C until biomarker testing was ready to be initiated. Assays were performed at the Harvard Medical School Clinical and Translational Science Award core laboratory at MGH. Plasma 25OHD (combined D_2_ and D_3_) levels were measured by enzyme-linked immunoabsorbent assay, using commercially available kits (Abbott Laboratories, Abbott Park, IL). Intra- and inter-assay coefficients of variation were both <10 %.

### Clinical data collection

The MGH electronic medical records system used abstract baseline demographic information, including (1) age, (2) sex, (3) race, (4) body mass index (BMI), and (5) type of patient (surgical vs. medical). To obtain in-hospital mortality data within 90-days of ICU admission, individual electronic medical records were reviewed. For patients discharged alive from the hospital and who continued to receive care within the Partners Healthcare network (which includes MGH and its affiliates), individual electronic outpatient medical records were reviewed to document all-cause mortality within 90 days of ICU admission. All individual records were cross-referenced with the Social Security Death Index Master File to finalize 90-day mortality cases.

### Statistical analysis

Descriptive statistics were tabulated for the analytic cohort. Continuous data were reported as means with standard deviations (SDs), and categorical values were expressed as proportions. To graphically represent the relationship between 25OHD levels and DCCI as well as APACHE II, we constructed locally weighted scatterplot smoothing (LOWESS) curves. LOWESS is a type of nonparametric regression, which summarizes a relationship between two variables in a fashion that initially relies on limited assumptions about the form or strength of the relationship [32]. The rationale and methods underlying the use of LOWESS for depicting the local relationship between measurements of interest across parts of their ranges have previously been described [33]. To investigate the association of 25OHD levels with 90-day mortality, we performed logistic regression analyses while controlling for biologically plausible covariates—as such, we developed two main models: the first controlled for (1) age, (2) sex, (3) race, (4) BMI, (5) type of patient, and (6) DCCI, while the second controlled for (1) age, (2) sex, (3) race, (4) BMI, (5) type of patient, and (6) APACHE II score.

Receiver operating characteristic (ROC) curves were constructed to assess the areas under the curve (AUC) for the ability of each predictive model to correctly identify survivors vs. non-survivors. An AUC of 0.5 suggests no predictive value, 0.7–0.8 suggests good predictive value, and >0.8 suggests excellent predictive value. Four different models were tested for their ability to predict 90-day mortality in the analytic cohort, namely (1) DCCI, (2) DCCI + 25OHD, (3) APACHE II, and (4) APACHE II + 25OHD. 25OHD levels were categorized into nationally accepted threshold levels of <10 ng/mL, 10–19.9 ng/mL, 20–29.9 ng/mL, and ≥30 ng/mL. Recent evidence suggests that these thresholds may be relevant and applicable to hospitalized and critically ill patients as well [34–36]. We assigned all plausible serial permutations of the sub-score values of the DCCI and APACHE II (0–6) to each 25OHD category in order to derive the highest AUC value. Serial number scores were assigned since the existing literature suggests that there is a near inverse linear association between 25OHD levels and adverse outcomes in hospitalized patients [34–36]. The final AUC values were compared for equality using the methodology described elsewhere [37]. Moreover, optimal cut-points were determined from the ROC curves by identifying scores that provided the highest cumulative value for sensitivity and specificity.

In a previous study [25], our group demonstrated that 100 ICU patients provided sufficient power to detect a meaningful association between admission 25OHD level as well as APACHE II score and 90-day mortality. Therefore, we assumed that the current analytic cohort size (*n* = 310) would be adequately powered to undertake the study objectives. All analyses were performed in STATA 13.0 (StataCorp LP, College Station, TX). A two-tailed *p* < 0.05, and any 95 % confidence interval not spanning 1, was considered statistically significant for all analyses.

## Results

**Fig. 1:**
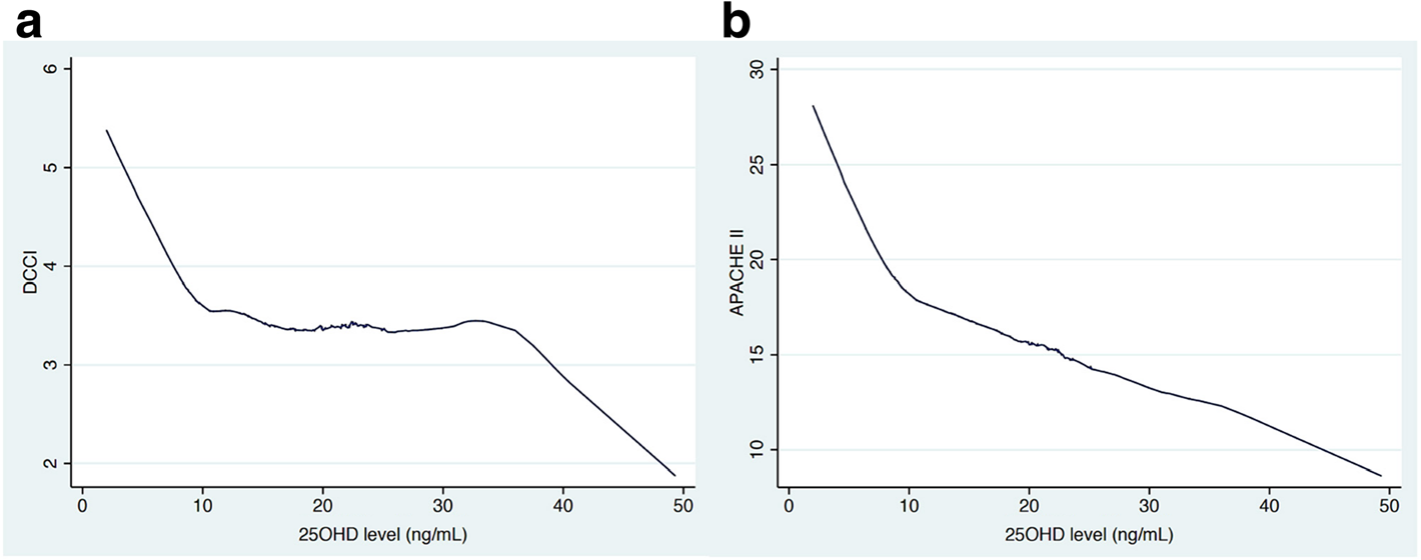
Locally weighted scatterplot smoothing (LOWESS) curve analysis of the relationship between vitamin D status and severity of illness scores. DCCI = Deyo-Charlson Comorbidity Index; 25OHD = 25-hydroxyvitamin D; APACHE II = Acute Physiology and Chronic Health Evaluation II. LOWESS curve analysis demonstrates a steep, near inverse, relationship between DCCI (**a**) as well as APACHE II (**b**) and 25OHD levels from 0 to 10 ng/mL. Beyond 25OHD levels of 10, there is progressive flattening of the curve

Three hundred and ten patients comprised the analytic cohort. All blood samples were obtained within 24 h of ICU admission, with 72 % of samples being obtained within 6 h of admission to the ICU. The general characteristics of the study cohort, stratified by 90-day survivors vs. non-survivors, are shown in Table 1. LOWESS curve analysis demonstrated a near inverse relationship between DCCI as well as APACHE II and 25OHD levels between 0 and 10 ng/mL (Fig. 1). Between 25OHD levels of 10 and 30 ng/mL, there was significant flattening of the curve. Multivariable logistic regression analyses including either DCCI or APACHE II score as a covariate demonstrated an inverse association between 25OHD levels and 90-day mortality in both models (OR 0.84; 95 % CI 0.79–0.91 and OR 0.86; 95 % CI 0.80–0.93, per 1 ng/mL, respectively). DCCI was independently associated with 90-day mortality (OR 1.32; 95 % CI 1.12–1.57), as was APACHE II (OR 1.14; 95 % CI 1.08–1.21).

**Table 1:** General characteristics of the study cohort (*n* = 310) stratified by 90-day survivors and non-survivors

Variable	90-day survivors (*n* = 251)	90-day non-survivors (*n* = 59)	*P* value
Age (years)	67 ± 14	69 ± 18	0.43
Sex (%)			0.15
Female	39	49	
Male	61	51	
Race (%)			*<0.001*
Non-white	5	32	
White	95	68	
BMI (kg/m^2^)	28 ± 7	30 ± 8	0.08
Type of patient (%)			*<0.001*
Surgical	76	43	
Medical	24	57	
25OHD (ng/mL)	20 ± 14	12 ± 7	*<0.001*
APACHE II	14 ± 7	24 ± 9	*<0.001*
Comorbidities (%)			0.21
Cardiovascular	92	97	
Pulmonary	24	46	
Renal	35	44	
Hepatic	5	12	
Sepsis	38	51	
Trauma	20	36	
DCCI	3 ± 2	5 ± 3	*<0.001*

Continuous data were reported as means with standard deviations (SDs), medians with interquartile ranges (IQRs), and categorical values were expressed as proportions. Body mass index = BMI, Acute Physiology and Chronic Health Evaluation II = APACHE II; Deyo-Charlson Comorbidity Index = DCCI; intensive care unit = ICU

P-values in italics represent statistically significant results

**Fig. 2:**
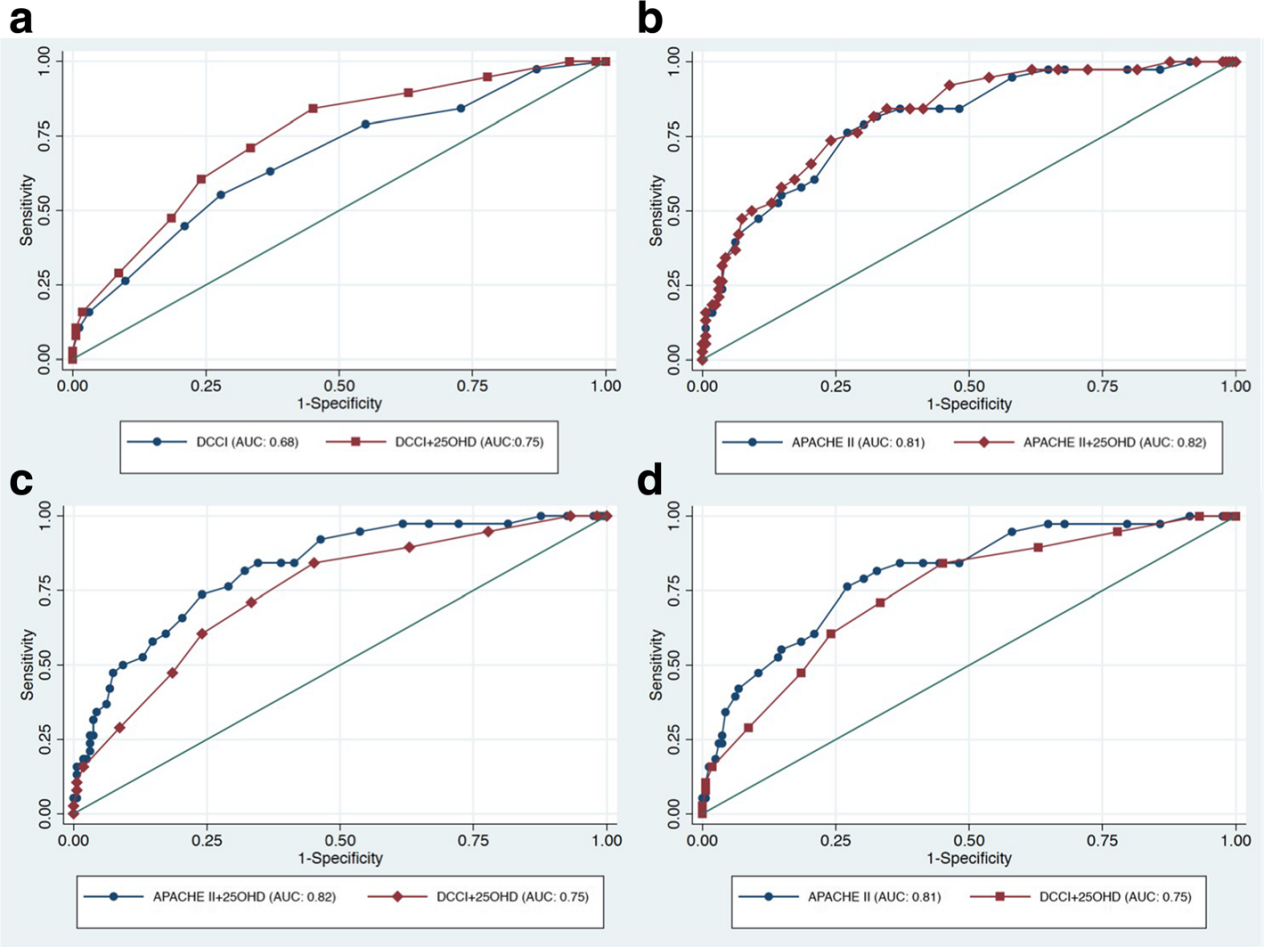
Receiver operating characteristic (ROC) curves and area under the curves (AUCs). DCCI = Deyo-Charlson Comorbidity Index; 25OHD = 25-hydroxyvitamin D; APACHE II = Acute Physiology and Chronic Health Evaluation II. **a** DCCI vs. DCCI + 25OHD (*p* < 0.001). **b** APACHE II vs. APACHE II + 25OHD (*p* < 0.001). **c** APACHE II + 25OHD vs. DCCI + 25OHD (*p* = 0.12). **d** APACHE II vs. DCCI + 25OHD (*p* = 0.04)

Initial score assignment for 25OHD thresholds was 0 for levels ≥30 ng/mL (since this is widely regarded as a “normal” level), 1 for levels 20–29.9 ng/mL, 2 for levels 10–19.9 ng/mL, and 3 for levels <10 ng/mL. The respective threshold scores were added to each DCCI and APACHE II score and ROC curves were generated to assess the AUCs (Fig. 2). Alternative score assignments did not result in either a statistically different, clinically meaningful, or biologically plausible difference in the calculated AUCs for DCCI + 25OHD or APACHE II + 25OHD. Moreover, based on the observed relationship between 25OHD and DCCI as well as APACHE II on LOWESS curve analysis, we also generated models where 25OHD levels ≥30 ng/mL designated a score of 0, levels 10–29.9 ng/mL were designated a score of 1, and levels <10 ng/mL were designated a score of 3. Again, this alternative scoring designation did not materially change any previously obtained results. As such, for the final models, the initial scoring assignment was retained. AUC for 25OHD alone was 0.75 (95 % CI 0.67–0.83). On the other hand, AUC for DCCI alone was 0.68 (95 % CI 0.59–0.77), and improved to 0.75 (95 % CI 0.66–0.83) with the addition of 25OHD threshold scores (*X*^2^ = 19.1, *p* < 0.001). AUC for APACHE II alone was 0.81 (95 % CI 0.73–0.88) and increased slightly to 0.82 (95 % CI 0.75–0.89) with the addition of 25OHD threshold scores (*X*^2^ = 11.4, *p* < 0.001). There was no difference between the AUC for 25OHD alone vs. DCCI + 25OHD (*X*^2^ = 0, *p* = 0.97). And while there was a significant difference between the AUC for DCCI + 25OHD vs. APACHE II + 25OHD (*X*^2^ = 4.4, *p* = 0.04), there was no difference in the AUC for DCCI + 25OHD vs. APACHE II alone (*X*^2^ = 2.38, *p* = 0.12).

The optimal cutoff value for 25OHD alone was ≥3 (i.e., <10 ng/mL), with a sensitivity of 45 %, specificity of 91 %, and accuracy of 68 %, while the optimal cutoff value for DCCI alone was ≥4, with a sensitivity of 63 %, specificity of 63 %, and accuracy of 63 %. On the other hand, the optimal cutoff value for DCCI + 25OHD was ≥5, with a sensitivity of 84 %, specificity of 55 %, and accuracy of 70 %. The optimal cutoff value for APACHE II was ≥12, with a sensitivity of 95 %, specificity of 42 %, and accuracy of 69 %, while the optimal cutoff value for APACHE II + 25OHD was ≥15, with a sensitivity of 92 %, specificity of 54 %, and accuracy of 73 %.

## Discussion

In this retrospective, cohort study, we investigated whether the addition of 25OHD levels to the DCCI at initiation of care improved 90-day mortality prediction (vs. the DCCI alone) in ICU patients and we compared these findings to the gold standard of APACHE II assessments. We demonstrated that the addition of 25OHD levels significantly improved the AUC for the DCCI, but did not materially affect the predictive capability of the APACHE II. Moreover, our results suggest that the DCCI + 25OHD model may perform as well as the APACHE II alone for predicting 90-day mortality in ICU patients. However, given the retrospective nature of our study, the real-time utility of DCCI + 25OHD over the APACHE II requires further investigation.

Previous studies have reported AUCs for the APACHE II method, which demonstrates excellent discrimination between survivors and non-survivors of critical illness (AUC >0.80) [4, 38, 39]. AUC for the APACHE II ROC in our study is consistent with what has been reported in the existing literature. Despite its clinically acceptable predictive value, APACHE II score calculations can be cumbersome if an appropriate infrastructure is not in place. Although most commercially available electronic medical record (EMR) systems automatically calculate the APACHE II score, these products may be cost-prohibitive, especially for smaller healthcare entities [40]. And without a robust EMR, APACHE II calculations must be performed manually by healthcare providers, which can be time consuming and therefore prone to low compliance. Furthermore, APACHE II scores may provide prognostic information too late after ICU admission. Since patients need to be admitted to the ICU for at least 24 h before a score can be calculated, the APACHE II does not help with critical decision-making for patients and families at the outset of critical illness. Indeed, many patients and families struggle with decisions to prolong medical therapy and/or undergo potentially painful as well as invasive procedures on admission to the ICU. As such, simple and timely predictive models for ICU-related mortality have the potential to assist clinicians in delivering more patient-centered, higher quality, and cost-effective care.

In 1987, Charlson et al. developed a weighted index, which took into account both the number and severity of various comorbid diseases; the purpose of the index was to develop a 10-year mortality prediction tool for the general population [6]. In its original form, the Charlson Comorbidity Index relied on patient interviews or chart review to classify comorbid diseases. Later, Deyo et al. adapted it to accommodate the use of the International Classification of Disease, 9th Edition, Clinical Modification (ICD-9-CM) to calculate the DCCI from administrative databases [9]. Other modifications to the original Charlson Comorbidity Index have included the use of alternate lists of specific diagnoses for comorbidities [15], re-assigning comorbidity weights [16, 17], and updating to the ICD-10-CM codes from ICD-9-CM codes [16, 18]. Recently, an increasing number of publications in acute care medicine have used the DCCI to risk-adjust for severity of illness [34–36, 41, 42]—indeed, mounting evidence suggests that the DCCI may have excellent in-hospital, 30-day, and 1-year mortality prediction in hospitalized patients [43]. Regarding the ICU, DCCI alone appears to have low predictive value (which is also observed in the present study) [41], but in combination with administrative data that reflect acute physiological processes (e.g., admission diagnosis, need for mechanical ventilation, initiation of renal replacement therapy), the AUC of the modified DCCI may be equivalent to the APACHE II [44]. While such adjustments are helpful for research purposes, they do not apply to real-time decision-making at the outset of critical illness. The use of non-administrative data, such as readily available or easily measured biomarkers, in conjunction with the DCCI has been reported to improve mortality prediction in dialysis-dependent renal failure patients [20] but until now has largely remained unexplored in the ICU.

Our study suggests that using easily obtained biomarker data, more specifically 25OHD levels at initiation of care, may significantly improve the predictive value of the DCCI for 90-day mortality in ICU patients, potentially making it a feasible alternative to the APACHE II score, especially when the necessary acute physiologic assessments are not readily available. Indeed, several studies suggest that 25OHD levels at initiation of critical care are inversely associated with mortality [25, 29, 45]. While vitamin D has traditionally been thought to play a vital role in bone [46], cardiac [47, 48], and muscle health [49], primarily by maintaining calcium balance, recent data support a more pleiotropic effect of vitamin D on general health [46]. With regards to critical illness, vitamin D plays a major role in maintaining immune heath. Recent studies have demonstrated that cells of the innate and adaptive immune system express the vitamin D receptor. Low 25OHD levels are associated with depressed macrophage phagocytosis, attenuated chemotaxis, and proinflammatory cytokine production [50]. Macrophages activated through the vitamin D receptor by 1,25-dihydroxyvitamin D (the most hormonally active vitamin D metabolite) upregulate expression of cathelicidin [51]. Cathelicidins are endogenous antimicrobial peptides that are active against a broad spectrum of infectious agents, such as bacteria, viruses, fungi, and mycobacteria [52]. Moreover, cathelicidins are highly expressed by epithelial cells at natural barrier sites (e.g., skin, lungs, gut) and may represent an important first line of defense for the innate immune system [53]. In addition, vitamin D is important for the interferon-γ-dependent T cell responses to infection [54] and therefore may play an important role in preventing immunoparalysis [55]. And finally, in animal models, vitamin D supplementation has been shown to improve coagulation variables and inhibit endotoxemia [56–58].

It is important to note that at present, it is not standard practice in most institutions worldwide to routinely obtain 25OHD levels in ICU patients. Traditionally, this has been due to a lack of evidence suggesting that assessment of vitamin D status might be helpful in critically ill patients; however, recent evidence supports the notion that 25OHD may be an important biomarker in this patient cohort [30, 35, 36]. Other major barriers to readily assessing vitamin D status in hospitalized patients worldwide have been the lack of in-house laboratory equipment, batched processing protocols, and the cost of the 25OHD assays themselves. Given the intricacies of accurately measuring steroid hormone levels, many facilities outsource such measurements, and as such, results may take several days to be finalized. Similarly, if the overall volume of testing is insufficient, many facilities process samples at dedicated time intervals—which may present a delay in obtaining results for bedside clinical decision-making. Led by the US National Institute of Standards and Technology and the National Institutes of Health, a global Vitamin D Standardization Program (VDSP) has been launched to assist facilities in ensuring the quality of their in-house assays and results [59–62]. Moreover, the recent availability of various point-of-care (POC) vitamin D testing devices has now made it feasible to obtain 25OHD levels to allow for real-time, bedside clinical decision-making [63–65]. These devices require very small amounts of blood (some are similar in design to POC glucose testing units for diabetics), are very affordable, and provide results within 10 min.

Although we present intriguing evidence regarding the addition of 25OHD and the DCCI to significantly improve mortality prediction in ICU patients, it is important to be mindful of potential limitations of our work. Retrospective cohort studies such as this cannot establish causation, but it can highlight the existence or absence of associations and thereby direct future research. Furthermore, observational studies may be limited by the lack of a randomly distributed exposure. And despite adjustment for the multiple potential confounders within the DCCI and APACHE II, there may still be residual confounding that contributes to the observed differences in survival. More specifically, vitamin D status may simply be a reflection of the overall health of patients, for which we may be unable to fully adjust. We were also unable to adjust for immobilization, lack of sun exposure, and vitamin D supplementation over the study period. In addition, 25OHD was only assessed within 24 h of ICU admission and may not completely represent vitamin D status at the outset of critical illness (since 25OHD levels may be influenced by inflammatory responses, fluid loading, and renal wasting of albumin as well as vitamin D binding protein) [30]. Moreover, changes in vitamin D status over the course of the study period were not assessed. The results of our study also may not be generalizable, since patients from only one institution and from only surgical or medical ICUs were included in the analysis (i.e., excluded patients from the neurosciences, cardiac, and burn ICUs). We also had a limited sample size (*n* = 310), which may further impact the generalizability of our findings. Despite these limitations, the biological plausibility of vitamin D status as a determinant of survival after critical illness is undeniable. Interestingly, 25OHD levels alone had a higher AUC than the DCCI; however, it is unlikely that clinicians would be willing to make key decisions based on a single biomarker and ignore the potential impact of chronic illness on critical care outcomes. Fortunately, the addition of vitamin D status to the DCCI maintains the observed AUC of 25OHD alone and allows for a prediction model that is as accurate as APACHE II alone. As such, the results of our study warrant further investigation to confirm these findings in larger datasets and to conduct future prospective studies.

## Conclusions

We confirm previous studies, which have suggested that total 25OHD levels upon ICU admission are inversely associated with the risk of mortality in the post-acute care setting. In addition, we present novel data that 25OHD levels on admission to the ICU may significantly improve the predictive value of the DCCI for 90-day mortality in critically ill patients. This may be particularly helpful in making clinical decisions at initiation of care in the ICU or in settings where prediction scores heavily based on physiologic assessments may not be readily available. Further prospective studies are needed to validate our findings and to determine whether optimizing vitamin D status in surgical ICU patients may confer survival benefit from critical illness.

## Availability of supporting data

Our Institutional Review Board proposal for this study specifically states that data will not be shared with any party outside of the Partners Healthcare Network; our database will not be available.

## Abbreviations

25OHD, 25-hydroxyvitamin D; APACHE II, Acute physiology and Chronic Health Evaluation score II; AUC, area under the curve; BMI, body mass index; DCCI, The Deyo-Charlson Comorbidity Index; EMR, Electronic medical record; ICU, intensive care unit; LOWESS, locally weighted scatterplot smoothing; MGH, Massachusetts General Hospital; OR, odds ratio; POC, point of care; ROC, receiver operating curve; SD, standard deviation; STATA, StataCorp LP; VDSP, Vitamin D Standardization Program
